# Supplementary material for: Air and surface contamination in non-health care settings among 641 environmental specimens of 39 COVID-19 cases
Source: PLoS Negl Trop Dis. 2020 Oct 9;14(10):e0008570. doi: 10.1371/journal.pntd.0008570 (PMC7577473; doi:10.1371/journal.pntd.0008570)
Supplement: S1 Appendix — (DOCX) [file pntd.0008570.s001.docx]

**Appendix Content**

[Appendix 1: Details regarding laboratory confirmation processes 1](#_Toc40730872)

[Test items 1](#_Toc40730873)

[Result judgment 1](#_Toc40730874)

[Table for COVID-19 sample collection and delivery 2](#_Toc40730875)

[Appendix 2: Definition 3](#_Toc40730876)

[Asymptomatic infected persons (asymptomatic cases in short) 3](#_Toc40730877)

[Severity of the disease 3](#_Toc40730878)

# Appendix 1: Details regarding laboratory confirmation processes

## Test items

SARS-CoV-2 nucleic acid determination (RT-PCR).

The SARS-CoV-2 primers and probes for ORF1ab and N gene regions are recommended.

**Target 1 (ORF1ab):**

Forward primer (F): ccctgtgggttttacacttaa

Reverse primer (R): acgattgtgcatcagctga

Fluorescent probe (P): 5 '- fam-ccgtctgcggtatgtggaaggtatgg-bhq1-3'

**Target 2 (N):**

Forward primer (F): gggggaacttcctgctagaat

Reverse primer (R): cagacatttgctcacgtg

Fluorescent probe (P): 5 '- fam-ttgctgctgcttgacagatt-tamra-3'

For nucleic acid extraction and real-time RT-PCR reaction system and reaction conditions, please refer to the kit instructions provided by relevant manufacturers.

## Result judgment

Negative: no CT value or CT ≥ 40.

Positive: CT value < 37, could be reported as positive.

Gray area: CT value is between 37-40, it is recommended to repeat the experiment. If the repeat result CT value is less than 40, the amplification curve has obvious peak, the sample is judged as positive, otherwise it is negative.

Note: if commercial kits are used, the instructions provided by the manufacturer shall prevail.

## Table for COVID-19 sample collection and delivery

Unit responsible for sample delivery (stamp): _______________________

Date of sample delivery: __________________

Person responsible for sample delivery: ______________

| Specimen number | Specimen type | Name | Sex | Age | Date of onset of the illness | Date of medical consultations | Sampling date | Clustered case or not | Date of testing | RT-PCR | | Homology of gene sequence* | | Note |
| --- | --- | --- | --- | --- | --- | --- | --- | --- | --- | --- | --- | --- | --- | --- |
|  |  |  |  |  |  |  |  |  |  | Reagent manufacturer | Target gene | Generation | Deep sequencing |  |
|  |  |  |  |  |  |  |  |  |  |  |  |  |  |  |
|  |  |  |  |  |  |  |  |  |  |  |  |  |  |  |
|  |  |  |  |  |  |  |  |  |  |  |  |  |  |  |
|  |  |  |  |  |  |  |  |  |  |  |  |  |  |  |
|  |  |  |  |  |  |  |  |  |  |  |  |  |  |  |
|  |  |  |  |  |  |  |  |  |  |  |  |  |  |  |

Homology of gene sequence * is not necessarily to fill in, give clear indication of the specific target gene sequence/ whole gene sequence that have already completed, and its homology with novel coronavirus. “Clustered case or not” fill yes or no.

# Appendix 2: Definition

## Asymptomatic infected persons (Asymptomatic cases)

The asymptomatic persons infected with COVID-19 virus (hereafter referred to as asymptomatic persons) refers to those who have no relevant clinical manifestations including clinically detectable signs or self-perceived symptoms such as fever, cough, or sore throat, but who have tested positive for COVID-19 virus in respiratory specimens or other specimens. These individuals may be found through tracing of close contacts, investigation of clusters, and tracing of sources of infection.

## Severity of the disease

Severity of the disease includes 4 categories: mild, moderate, severe and critical. Mild cases were those who had mild symptoms and no sign of pneumonia on chest imaging. Moderate cases are those who had fever and respiratory symptoms and signs of pneumonia. Severe cases were those who meet any of the falling criteria: 1) Shortness of breath, RR ≥30times/min; 2) Oxygen saturation ≤93% at rest; 3) Alveolar oxygen partial pressure/fraction of inspiration O2 (PaO2/FiO2) ≤300 mmHg(1mmHg=0.133kPa). Critical cases are those who meet any of the falling conditions: 1) respiratory failure requiring mechanical ventilation; 2) Shock; 3) Patients combined with other organ failure needed ICU monitoring and treatment.
